# A new removable uterine compression suture (RUCS) as an effective treatment for postpartum hemorrhage without long-term uterine synechiae: a retrospective preliminary study

**DOI:** 10.1186/s12884-026-08805-y

**Published:** 2026-02-17

**Authors:** Havva Betul Bacak, Suleyman Salman, Serkan Kumbasar, Fatma Ketenci Gencer, Enes Serhat Coskun, Fatih Irice, Ecenur Celikoglu, Yagmur Aciyiyen, Deniz Ersoy, Rana Guven, Sebnem Atar, Turgut Ozbay

**Affiliations:** Department of Obstetrics and Gynecology, University of Health Sciences, Gaziosmanpasa Training and Research Hospital, Istanbul, Türkiye Turkey

**Keywords:** Removable uterine compression suture, Postpartum hemorrhage, Uterine atony

## Abstract

**Objectives:**

Uterine compression sutures have proven to be a valuable and safe option in the control of Postpartum hemorrhage. To avoid complications related to uterine compression sutures (uterine necrosis, synechia, and pyometra), we assess the efficacy of removable uterine compression suture (RUCS) for primary postpartum hemorrhage (PPH) and evaluate its effectiveness.

**Study design:**

This retrospective preliminary study was conducted at a tertiary referral hospital between January 2020 and November 2024, including patients diagnosed with postpartum hemorrhage who required compression sutures. Demographic characteristics of the patients who underwent RUCS and postpartum clinical parameters were determined.

**Results:**

Bleeding was successfully controlled in all 11 patients (100%). A mild, localized ecchymosis occurred at the unilateral suture exit site and resolved spontaneously. No intrauterine synechiae were detected in any of the 11 patients at 6-month hysteroscopic evaluation. Fertility outcomes were available for six women, five of whom achieved subsequent pregnancies.

**Conclusion:**

In this study, we present a novel removable compression suture that appears to be effective, simple, and rapid in application, with the potential to reduce serious complications; however, further evaluation is warranted.

## Introduction

 Postpartum hemorrhage (PPH) is the most important cause of maternal mortality and morbidity in developing countries [1]. The most common cause of primary PPH is uterine atony (UA) [2]. The majority of PPH is controlled by conservative management such as uterine fundal massage or bimanual uterine compression, various uterotonic agents, intrauterine gas tamponade, intrauterine balloon catheter [3, 4]. However, in cases where relatively non-invasive methods have failed, more invasive treatments must be used including uterine artery ligation, iliac artery ligation, or hysterectomy. Emergency peripartum hysterectomy rate is between 0.020% and 0.509%, respectively [5]. Hysterectomy is a life-saving operation under emergency conditions, but it has important consequences including unexpected loss of fertility, significant psychological trauma, and is also a cause of serious morbidity in terms of urogenital system injuries [6, 7].

Since the publication of the first B-lynch technique [8], different uterine compression sutures have been described. These suture techniques have been applied as an alternative to hysterectomy. However, some women may not respond to B-Lynch sutures and may still require hysterectomy [9]. Uterine compression sutures have proven to be a valuable and safe option in the control of PPH [10]. Some complications have occurred over the years [11]. It has been reported that uterine synechiae is a complication observed in 18–54% of compression sutures and decreases fertility [12, 13]. In addition, although rare, serious complications such as uterine necrosis have been reported [14, 15]. The removable nature of removable uterine compression suture (RUCS) allows the suture to remain in place only briefly, which may help minimize prolonged myometrial compression, thereby reducing the likelihood of ischemia-related necrosis, intrauterine synechiae, and secondary infection that have been reported after permanent compression sutures. In our institution, the RUCS technique was introduced primarily in response to concerns regarding intrauterine synechiae formation reported after permanent uterine compression sutures. The main rationale was to minimize prolonged endometrial and myometrial compression that may contribute to adhesion development, while maintaining effective hemostasis and preserving uterine function. Following initial clinical experience, RUCS was incorporated into the institutional postpartum hemorrhage management algorithm as a uterus-preserving surgical option in selected refractory cases.

In this study, we discuss the efficacy and safety of the new removable uterine compression suture in reducing the possible detrimental consequences of conventional compression sutures (synechiae, uterine necrosis) and in the management of PPH.

## Materials and methods

Eleven patients diagnosed with postpartum hemorrhage between January 2020 and November 2024 in Gynecology and Obstetrics Clinic of Gaziosmanpasa Training Research Hospital were included in the study. This study was approved by the Clinical Research Ethics Committee of Gaziosmanpaşa Training and Research Hospital (Approval No: 74, Date: 28.05.2025). All procedures were carried out in accordance with the Declaration of Helsinki. The trial was registered at ClinicalTrials.gov (Identifier: NCT07115355).

During the study period, all women who developed primary postpartum hemorrhage requiring surgical intervention were screened. Patients who did not respond to standard medical management and whose bleeding persisted despite bilateral uterine artery ligation were evaluated for RUCS application according to the institutional stepwise management algorithm. Eligible patients were identified retrospectively using the institutional electronic medical record system and delivery database. Patients who underwent RUCS application were consecutively included in the study.

Patients were included if postpartum hemorrhage occurred secondary to fundal uterine atony that did not respond to standard medical management (uterine massage, oxytocin, carbetocin, methylergonovine, tranexamic acid, fibrinogen). Pure lower-segment atony without fundal involvement was excluded because intrauterine balloon tamponade was not part of the institutional protocol. Pure lower-segment atony was clinically defined as the presence of adequate fundal uterine contraction with persistent bleeding originating from a poorly contracted lower uterine segment, as determined by bimanual uterine examination and intraoperative assessment. When bleeding persisted, bilateral uterine artery ligation was performed as the first surgical step; RUCS was subsequently applied in cases refractory to arterial ligation. Patients with PPH due to uterine rupture, extensive genital tract lacerations, or placenta accreta spectrum requiring primary hysterectomy were excluded. Women who underwent hysterectomy without prior RUCS were excluded from clinical and reproductive follow-up analyses. Immediate hysterectomy without prior RUCS application was performed exclusively in cases with placenta accreta spectrum (PAS), in accordance with institutional management protocols. These patients were excluded from clinical and reproductive follow-up analyses because RUCS-related uterine outcomes could not be evaluated.

Demographic characteristics, including age, gravida, parity, gestational age, and mode of delivery, were recorded. Postpartum hemorrhage was diagnosed when estimated blood loss exceeded 1000 mL, consistent with World Health Organization (WHO) and American College of Obstetricians and Gynecologists (ACOG) criteria. Blood loss was clinically assessed using operative records, suction volumes, and visual estimation as recommended in major guidelines.

The stepwise algorithm for PPH management in our institution was adapted from WHO recommendations. All patients first received standard care consistent with the WHO EMOTIVE bundle—early recognition, uterine massage, uterotonics, tranexamic acid, fluid resuscitation, and continuous monitoring. Surgical intervention was initiated when these measures failed to control bleeding. Bilateral uterine artery ligation was routinely performed as the first surgical step, and RUCS was applied in all women whose bleeding persisted despite arterial ligation.

RUCS was introduced in our institution primarily in response to concerns regarding intrauterine synechiae formation reported after permanent uterine compression sutures. The institutional rationale was to minimize prolonged endometrial and myometrial compression while maintaining effective hemostasis and preserving uterine function. Following an initial familiarization period among experienced obstetric surgeons, the technique was incorporated into the stepwise postpartum hemorrhage management algorithm. Given its standardized and reproducible nature, no prolonged learning curve was required, and the procedure was performed by trained senior obstetricians.

**Fig. 1:**
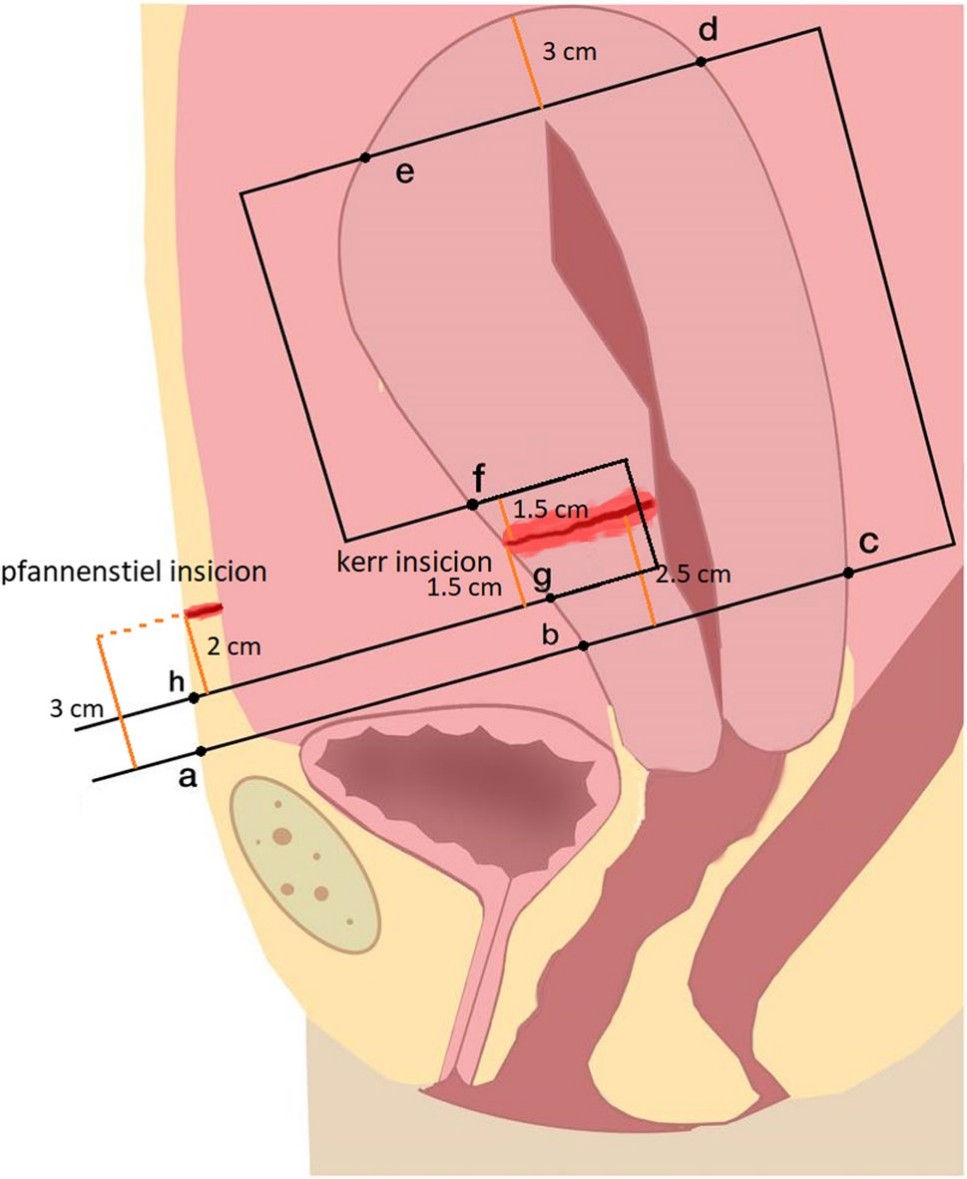
Schema of removable uterine compression suture (RUCS). Point a: the point where the suture passes through the skin. Point b: the point where the suture passes through the lower segment of the anterior aspect of the uterus, Point c: the point at which the lower segment of the uterus emerges from the posterior face, Point d: the point where the suture passes through the posterior region of the uterus fundus, Point e: the point where the suture exits from the uterus fundus anterior, Point f: the point where the suture enters the myometrium at the anterior face of the uterus again, Point g: the point at which the suture exits myometrium, Point h: the point where the suture comes out of the skin

**Fig. 2:**
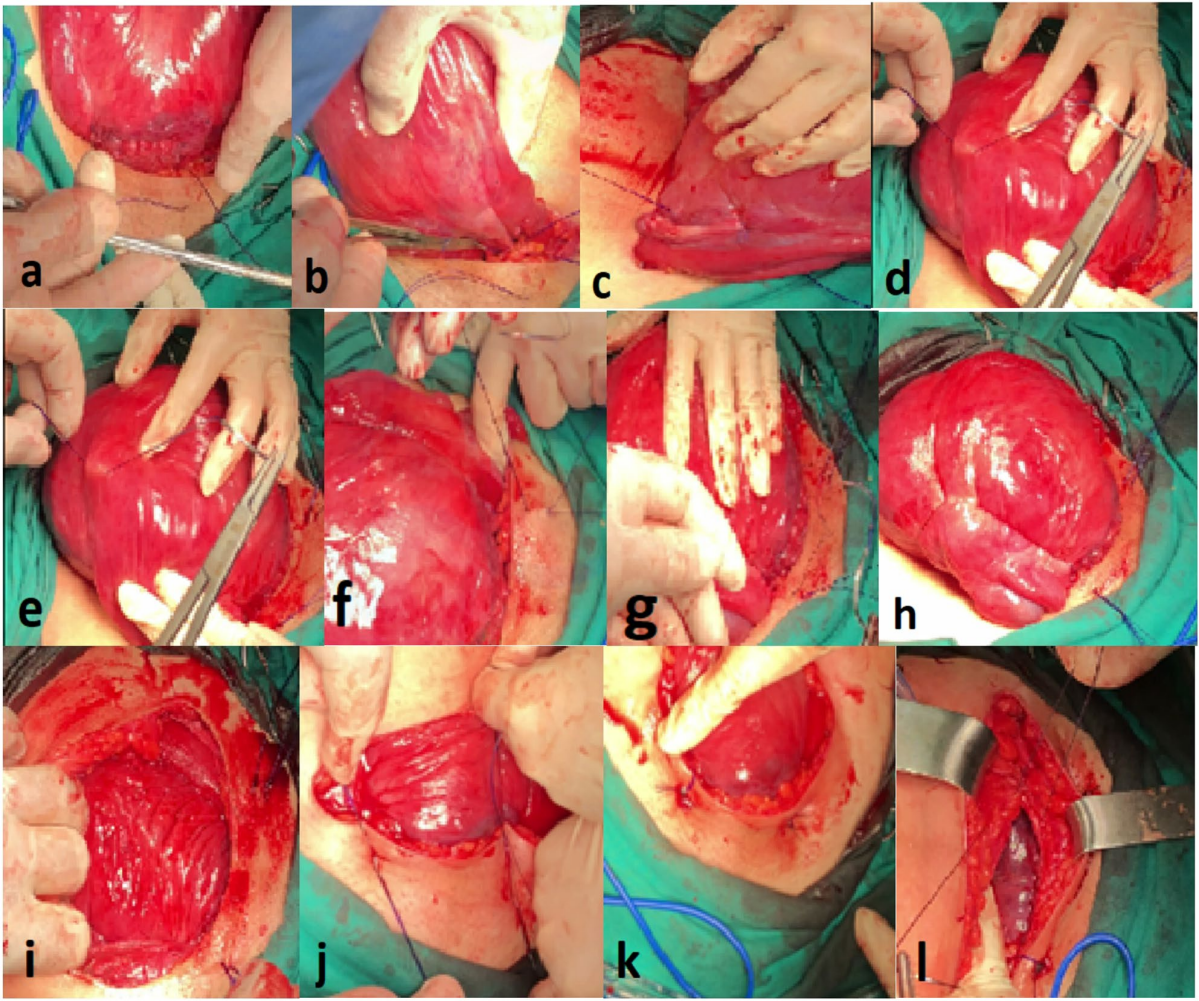
Application of removable uterine compression suture (RUCS) during surgery. **a** First needle pass through the anterior lower uterine segment. **b** The suture is pulled through and positioned on the uterine surface. **c** The strand is guided cranially along the uterus to plan the compression pathway. **d** The needle is advanced toward/over the fundus. **e** The maneuver is repeated on the contralateral side. **f** Additional seromuscular pass to complete the compression course. **g** The suture ends are tightened to achieve uterine compression. **h** Appearance of the uterus after tightening (effective compression). **i** Final compression configuration over the uterine surface. **j** Securing the suture while maintaining removability. **k** Removable end/loop left accessible for later removal. **l** Fascial closure at the end of the procedure

RUCS was placed using a monofilament polypropylene (Prolene) suture. Polypropylene was preferred over Vicryl because its monofilament structure results in lower tissue drag and bacterial adherence, and it allows easier atraumatic removal within 18–24 h—features desirable for a removable compression suture (Figs. 1 and 2).

In cases complicated by placenta previa, intrauterine balloon tamponade was applied specifically to control persistent bleeding originating from the placental implantation site in the lower uterine segment, whereas atony-related hemorrhage was managed with bilateral uterine artery ligation and RUCS (Schema 1).

**Schema 1:**
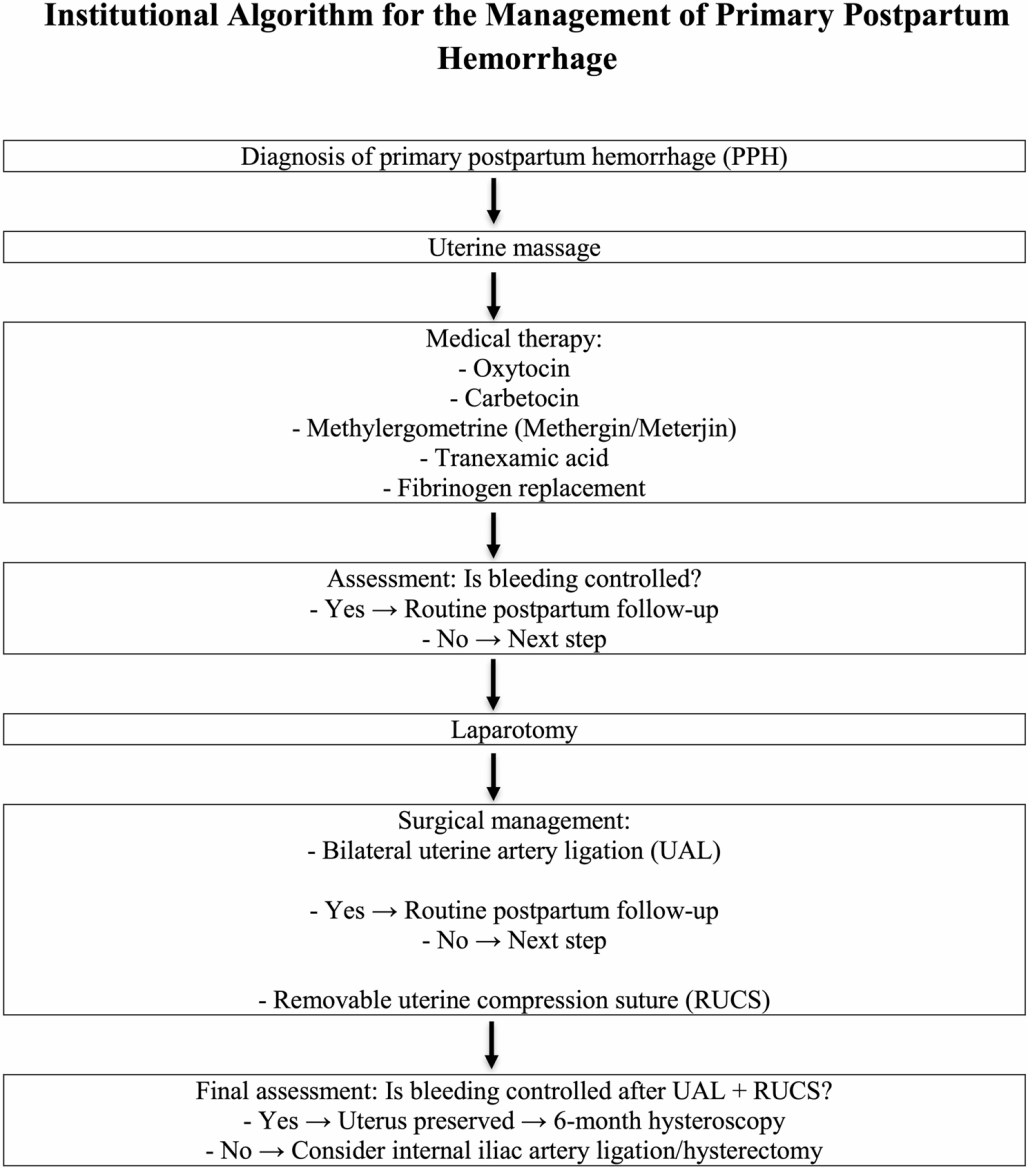
Stepwise algorithm for the management of primary postpartum hemorrhage, adapted from WHO recommendations and tailored to our institutional clinical practice

RUCS was performed either during CS or after vaginal delivery, with the latter requiring laparotomy. In patients undergoing cesarean section, the uterine incision was fully closed prior to RUCS placement. First, the bladder peritoneum was dissected off the lower uterine segment. A No. 1 polypropylene non-absorbable suture on a 70-mm round-bodied hand needle was then introduced through the abdominal skin, traversing the full thickness of the anterior abdominal wall approximately 3 cm above the inferior border of the pubic symphysis and approximately 3 cm inferior to the lower margin of the skin incision (Fig. 1 line a to b; Fig. 2 a). Because the uterus is typically soft and atonic in postpartum hemorrhage, full-thickness needle passage was easily achieved using a 70-mm round-bodied needle, and minor adjustment of needle curvature was performed when needed. The needle was subsequently passed through the lower uterine segment at a point 2.5 cm below the Kerr incision, directed from anterior to posterior (Fig. 1 line b to c; Fig. 2 b, c). It was then reinserted at the posterior uterine wall, ideally around 3 cm below the fundus, and a full-thickness fundal bite was obtained by passing the needle from posterior to anterior (Fig. 1 line d to e; Fig. 2 d). Next, the needle was introduced 2 cm above the Kerr incision, advanced deeply within the myometrium, and brought out 2 cm below the Kerr incision without approaching closer than 2 cm to the uterine artery, thus exiting through the lower uterine segment (Fig. 1 line f to g; Fig. 2 e). Finally, the needle was directed through the anterior abdominal wall, emerging 2 cm below the skin incision (Fig. 1 line g to h; Fig. 2 f, g, h), where the suture ends were secured. The same procedure was repeated on the contralateral side. The longitudinal limbs of the suture slid over the uterine fundus, thereby ante flexing the uterus. The two ends of the suture were then drawn tightly, and a double knot was tied under sufficient tension to achieve effective hemostatic compression. In RUCS, two longitudinal sutures are employed. The knots were in a position to be seen through the skin (Fig. 2). After the RUCS knots were secured externally, the rectus fascia was closed in the standard manner, followed by skin closure, while the externalized suture ends were left accessible for later removal. Approximately twenty-four hours after the procedure, depending on the patient’s hemodynamic stability, the knots were cut, and the sutures were removed by simple traction without anesthesia.

All patients who underwent RUCS were evaluated with office hysteroscopy at 6 months postpartum to assess intrauterine healing and detect possible synechiae. One hysteroscopy was video-recorded for documentation, while the remaining evaluations were documented as written reports in the hospital’s digital archive.

## Results

RUCS was performed in 11 women, corresponding to 0.8 per 1,000 births (0.08%). The distribution of PPH cases in the study population is presented in Schema 2. During the study period, there were 13,835 births in our institution, of which 277 were complicated by postpartum hemorrhage. Among these, 27 women required surgical intervention and all initially underwent bilateral uterine artery ligation as the first surgical step. In 16 of the 27 women requiring surgical intervention, adequate hemostasis was achieved after bilateral uterine artery ligation and no additional surgical procedure was required. 11 women did not respond to standard medical management and arterial ligation, and hemorrhage persisted. RUCS was subsequently applied in these 11 patients, who constitute the final study population.

**Scheme 2:**
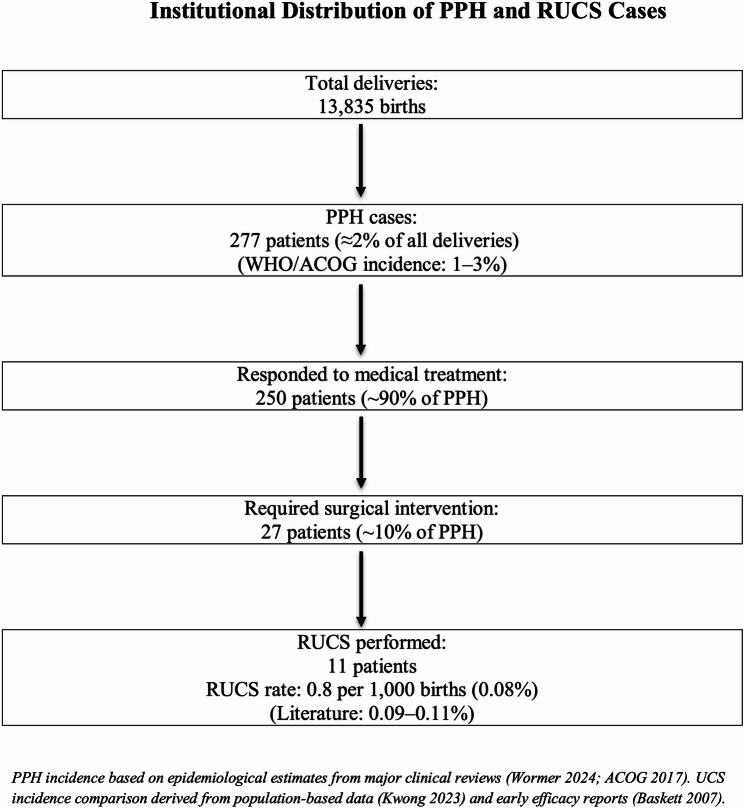
Distribution of postpartum hemorrhage (PPH) cases in the study population, including estimated PPH incidence, medical responders, surgical candidates, and RUCS frequency

In the case series, 7(63.6%) of 11 cases with PPH were delivered by cesarean section and 4 of them had vaginal delivery. The mean age of the patients was 29.72 ± 4.51. The mean gravida was 2.71 ± 0.9 and the mean parity was 1.72 ± 0.98, respectively. The mean gestational age of the patients was 37.36 ± 3.47 weeks (28w-41w). Among the causes of PPH, 9 (81.8%) patients had atony bleeding (4 due to prolonged labor, two due to macrosomic baby, one due to placenta previa + twin pregnancy, one due to polyhydramnios and one due to Placenta previa + fourth previous caesarean delivery), one case (9.09%) had placental abruption, and one case had placenta previa (Table 1). Bleeding control was achieved in 11 patients (100%) that underwent the suture process. Bilateral uterine artery ligation and RUCS were performed in all cases (Table 2). No alternative uterine compression sutures were used during the study period.

In the three placenta previa cases, RUCS was applied to control atony-related hemorrhage; however, persistent bleeding from the lower uterine segment prompted the additional use of balloon tamponade.

**Table 1:** Demographic characteristics and study results of RUCS patients

Cases	Age	Gravida/ parity	Gestation (weeks)	Vaginal delivery	Cesarean section	Intra-operative procedures -UAL -RUCS -Balloon tamponade	Average blood loss	Number of transfusions	Causes of postpartum hemorrhage	Time to suture removal (hour)	Time of Procedure (minute)	Complication	Office HSC (at 6 months)
1	31	G3p2	34		LSCS	Ual+RUCS + balloon tamponade	2000	6 prbc+6ffp	Atony (Placenta previa + twin pregnancy)	18	10	-	N
2	28	G3p2	28		Emergency LSCS	Ual+RUCS	2500	8prbc + 8 ffp	Decolman placenta	24	12	-	N
3	24	G2p1	38	Vaginal delivery		Ual+RUCS	2000	5prbc + 4 ffp	Atony (prolonged labor)	17	13	-	N
4	25	G2p1	41		LSCS	Ual+RUCS	2500	7prbc +7ffp	Atony (large baby)	22	11	-	N
5	29	G4p3	39		Emergency LSCS	Ual+RUCS	3000	8prbc +8ffp + 4 fibrinogen	Placenta previa	24	10	Local ecchymosis	N
6	28	G2p1	40	Vaginal delivery		Ual+RUCS	2500	6prbc+6ffp	Atony (prolonged labor)	18	12	-	N
7	34	G3p2	38	Vaginal delivery		Ual+RUCS	3000	6prbc +6ffp + 2 fibrinogen	Atony (prolonged labor)	18	15	-	N
8	36	G2p1	39		LSCS	Ual+RUCS	2000	5prbc +5ffp	Atony (polyhydroamnios)	20	12	-	N
9	32	G4p3	37		Emergency LSCS	Ual+RUCS+balloon tamponade	1500	3prbc + 3ffp	Atony (Placenta previa + fourth previous caesarean delivery)	18	10	-	N
10	37	G4p3	37		LSCS	Ual+RUCS	2500	6prbc + 5ffp+ 1fibrinogen	Atony (Macrosomia)	24	10	-	N
11	23	G1po	40	Vaginal delivery		Ual+RUCS	2000	4prbc + 4ffp+1fibrinogen	Atony (prolonged labor)	22	12	-	N

*LSCS* lower segment caesarean section, *PBC* packed blood cells, *FFP* fresh frozen plasma, *UAL* uterine artery ligation, *RUCS* removable uterine compression suture, *HSC* hysteroscopy, *N* normal

The mean blood loss was 2318 ± 440.69 cc (1500–3000 cc) and the mean transfusion applied to the patients was 5.8 ± 1.4 (3–8 units) of pRBC (packed red blood cells) and 5.63 ± 1.55 (3–8 units ffp) of fresh frozen plasma and 0.72 ± 1.21 (0–4 units) of fibrinogen. The indication for surgical intervention was based on persistent active bleeding despite standard medical management and uterine artery ligation, rather than absolute estimated blood loss values alone. The procedure lasted an average of 11.54 ± 1.49 min. Procedure time was calculated from abdominal entry to completion of RUCS placement. Using this standardized definition, operative times were comparable between vaginal delivery and cesarean section cases. Postoperatively, a mild local ecchymosis at the suture exit site was noted in one patient, which resolved spontaneously without requiring further intervention. The patients were discharged on average 4.3 ± 1.03 (3–6) days, and the mean time to suture removal was 20.45 ± 2.67 h. At the time of suture removal, a mild subjective reduction in suture tension was observed in most cases, consistent with early uterine involution; however, no recurrent bleeding was noted after removal. Intraoperative antibiotics (cefazolin 1gr) were started and continued for 5 days (Table 2).

No uterine synechiae were observed in any of the patients during the 6-month follow-up office hysteroscopy. No additional intrauterine pathological findings such as cavity distortion or abnormal endometrial lesions were observed. In patients with previous cesarean delivery, isthmocele-related findings were occasionally noted; however, these were not systematically documented and were not included in the formal analysis. During routine postpartum outpatient follow-up, no major procedure-related complications or adverse events were recorded between hospital discharge and the 6-month hysteroscopic evaluation (Table 2). No clinical or hysteroscopic evidence of anterior uterine wall adhesion or abnormal uterine fixation was observed during follow-up examinations.

All patients with available fertility outcomes were evaluated at 36 months postpartum. Fertility outcomes were available for six women: one conceived twice, three conceived once, and one had a spontaneous abortion followed by a normal ongoing pregnancy. One patient declined future fertility. The remaining five women were lost to reproductive follow-up after the 6-month hysteroscopy (Table 2).

**Table 2:** Baseline characteristics, clinical findings, and outcomes of women undergoing RUCS

Variable	Value
Number of patients	11
Age (years, mean ± SD)	29.72 ± 4.51
Gravida (mean ± SD)	2.71 ± 0.90
Parity (mean ± SD)	1.72 ± 0.98
Gestational age at delivery (weeks, mean ± SD)	37.36 ± 3.47 (28–41)
Mode of delivery	Cesarean: 7 (63.6%); Vaginal: 4 (36.4%)
Causes of PPH	Atony: 9 (81.8%) — prolonged labor (*n* = 4), macrosomia (*n* = 2), placenta previa + twin pregnancy (*n* = 1), polyhydramnios (*n* = 1), placenta previa + 4th CS (*n* = 1); Abruption: 1 (9.09%); Placenta previa: 1 (9.09%)
Mean blood loss (mL)	2318 ± 440.69 (1500–3000)
Transfusion requirements	pRBC: 5.8 ± 1.4 units (3–8); FFP: 5.63 ± 1.55 units (3–8); Fibrinogen: 0.72 ± 1.21 units (0–4)
Bilateral uterine artery ligation	Performed in all patients
Adjunctive interventions	RUCS in all patients; balloon tamponade additionally used in 3 placenta previa cases for lower uterine segment bleeding.
Bleeding control success rate	100% (11/11)
Procedure duration (minutes)	11.54 ± 1.49
Suture removal time (hours)	20.45 ± 2.67
Antibiotic regimen	Cefazolin 1 g perioperatively + 5-day postoperative course
Postoperative complications	One local ecchymosis; no uterine synechiae on 6-month hysteroscopy
Length of hospital stay (days)	4.3 ± 1.03 (3–6)
Subsequent fertility outcomes	Data available for 6 women: 1 patient conceived twice; 3 conceived once; 1 had one spontaneous abortion followed by a normal ongoing pregnancy; 1 declined future fertility
Lost to reproductive follow-up	5 women (after 6-month hysteroscopy)

Values are expressed as mean ± SD unless otherwise specified

## Discussion

There is a need for a uterine compression suture that eliminates uterine synechiae or other complications. In this respect RUCS can be an alternative for avoiding uterine synechiae. This preliminary study is the first showing RUCS has the potential to overcome synechiae after PPH compression sutures.

To date, no randomized controlled trial has been conducted to determine the best uterine compression suture for achieving hemostasis [16]. According to a previously published review including 11 original studies with a total of 109 women, the reported mean hemostasis rate of uterine compression sutures was 97% (103/109), with a range of 76–100%.^(17)^ In cases that did not respond to medical treatment in post-partum atony, different compression sutures were used to control PPH, including B-Lynch suture [8], Cho suture [17], Hayman suture [18], and Matsubara-Yano suture [19]. For postpartum hemorrhage predominantly originating from lower uterine segment atony, vaginal packing has also been reported as a simple and feasible uterus-preserving option [20]. Koh et al. used B-Lynch alone in 7 cases with UA, 6 required no additional suture, and 1 required hysterectomy when bleeding continued [2]. Ouahba et al. performed modified compression suture in the uterus of 20 patients, they stopped bleeding in 19 patients [21]. Success was achieved in 12 of 13 patients; only one case with disseminated intravascular coagulation (DIC) failed. Mostafa et al. used a compression suture without entering the uterine cavity [22].

High success and fertility preservation are key advantages of compression sutures, while uterine necrosis, synechiae, and pyometra are reported drawbacks [11, 14, 15]. Grotegut et al. reported that the B-Lynch suture caused erosion of the uterine wall [23]. Subsequently, uterine necrosis after B-Lynch suture has been widely described by various authors in the literature [14, 15, 24]. Some researchers have also reported pyometra and uterine synechia after the use of hemostatic square/compression sutures [25, 26]. In our study there is no major complication like synechiae or any other.

To reduce complications, Aboulfelah et al. (2014) introduced removable uterine compression sutures [27]. Aboulfelah et al. introduced a removable uterine compression brace suture in 15 PPH cases, 11 of which were due to uterine atony. Only one patient required hysterectomy due to rebleeding. Hemostasis was attributed to two mechanisms: reduced uterine artery flow from upward traction and compression of placental beds via uterine wall approximation. The suture, removed after 24 h, was suggested to lower the risk of synechiae and necrosis [27]. Zhang et al. used two types of removable uterine compression sutures in five cases: removable B-Lynch in two and Hayman in three. Sutures were removed vaginally, and bleeding was controlled in all cases [28]. Li and colleagues described a new ring uterine compression suture (RCS) in their series of 12 cases during cesarean Sect [29]. Li described a ring uterine compression suture (RCS) placed below the uterosacral ligament, passed through the cervix and abdominal wall, and tied externally on the pudendum. Bleeding was controlled in all cases without RCS-related complications. The authors suggested incorporating this method into first-line surgical management of PPH [29].

In our model, unlike the removable hayman and removable b-lynch sutures applied by Zhang et al., excessive uterine flexion of the uterus and the suture bringing the walls of uterus closer to each other play a role. Unlike Zhang’s and Li’s method, the strings can be easily removed from the skin with our described model. Unlike the Aboulfalah method, the needle is directly inserted from the posterior part of the uterus fundus to exit the anterior part of the fundus (Fig. 2line d to e) to prevent sutures from sliding over the uterus. In addition, a deep suture passes through the myometrium of the lower segment of the uterus (Fig. 2 line f to g) Bleeding was stopped in all 11 patients with RUCS. We believe that RUCS is simpler and more practical than any of the methods that previously reported.

In our institution, postpartum hemorrhage occurred in 277 of 13,835 births (2.0%), which is in line with epidemiological estimates reporting a 1–3% incidence in the general obstetric population [30, 31]. Among these women, 11 did not respond to medical therapy or bilateral uterine artery ligation and subsequently underwent RUCS. The incidence of RUCS in our cohort (0.8 per 1,000 births) falls within the population-based range reported for uterine compression sutures, which is approximately 0.7–1.0 per 1,000 births [32, 33]. In our institutional protocol, the indication for RUCS was not based solely on absolute estimated blood loss values, but rather on the presence of persistent active hemorrhage despite standard medical treatment and uterine artery ligation, as well as inadequate hemostatic control. Transfusion management was guided by institutional postpartum hemorrhage and massive transfusion protocols, with early balanced administration of red blood cells and plasma to maintain hemodynamic stability and prevent dilutional coagulopathy in patients with ongoing active bleeding.

Prolonged tension of the suture in the myometrium causes ischemia, hypoxia, and necrosis of the uterus. Ischemia and necrosis in the endometrium may cause uterine synechia, pyometra, and secondary infertility [34]. The temporary and removable nature of RUCS limits the duration of sustained endometrial compression, which may theoretically reduce ischemia-related endometrial injury and subsequent intrauterine adhesion formation compared with permanent compression sutures. We removed the removable, modified uterine compression sutures when the patient’s hemodynamic stability was fully improved or there was minimal vaginal bleeding. The removable nature of RUCS may limit the duration of sustained myometrial compression and prolonged ischemic exposure; however, early pressure-related tissue stress cannot be completely eliminated. The mean time to remove sutures was 20.45 ± 2.67 h. No bleeding was observed in any patient after removal of the suturs. No significant complications were observed during the puerperium, and all women returned to their normal menstrual cycles. A mild local ecchymosis was noted at the suture exit site in one patient and resolved spontaneously. Notably, no intrauterine synechiae were identified on 6-month hysteroscopic evaluation in any patient.

Several notable strengths characterize this study. All RUCS procedures were performed in a standardized manner by experienced obstetric surgeons, ensuring consistency in the application of the technique despite being performed by more than one operator. Systematic hysteroscopic evaluation at six months allowed objective assessment of intrauterine healing, which is rarely reported in compression suture studies. In addition, this series represents one of the earliest and more detailed evaluations of a removable uterine compression suture, with complete bleeding control achieved in all cases within this small cohort. Fertility outcomes were available for more than half of the women, providing preliminary insight into reproductive safety following RUCS. Finally, the surgical technique was described step-by-step and supported by illustrative figures, enhancing the reproducibility of the method.

However, certain limitations should be acknowledged. First, its retrospective and single-center design limits the level of control over potential confounders and reduces generalizability. The small sample size and the absence of a comparison group using conventional compression sutures preclude direct assessment of relative efficacy and do not allow definitive conclusions to be drawn. Postoperative pain outcomes could not be quantitatively evaluated because standardized pain scoring systems were not routinely documented due to the retrospective design. Future prospective studies should incorporate systematic pain assessment to better evaluate patient comfort following RUCS. Because of the retrospective study design, systematic prospective documentation of minor postpartum symptoms during the follow-up interval was limited. Furthermore, reproductive outcomes could only be assessed in six women, as five were lost to long-term follow-up. Reproductive follow-up was limited by the retrospective design of the study, loss of contact with some patients, and variability in future pregnancy planning, which restricted the availability of long-term fertility outcome data. Larger, prospective, multicenter, and comparative studies are needed to validate the safety and effectiveness of RUCS.

## Conclusions

In this preliminary study, we present a novel removable uterine compression suture technique. This technique does not exert prolonged tension on the myometrium, which may help reduce hypoxia-related tissue injury and potentially lower the risk of intrauterine synechiae or uterine necrosis in the postpartum period. Although the procedure appears to be simple to learn and feasible in emergency settings, definitive conclusions cannot be drawn from this preliminary study. Therefore, further large-scale, prospective, and comparative studies are required to validate the safety and effectiveness of RUCS in comparison with conventional compression sutures.

This study was conducted at Department of Obstetrics and Gynecology, University of Health Sciences Gaziosmanpasa Training and Research Hospital.

## Data Availability

All data generated and analyzed during this study are included in this published article. No additional datasets are available.
